# Inhibition of 2-AG hydrolysis differentially regulates blood brain barrier permeability after injury

**DOI:** 10.1186/s12974-018-1166-9

**Published:** 2018-05-14

**Authors:** Justin R. Piro, Georgette L. Suidan, Jie Quan, YeQing Pi, Sharon M. O’Neill, Marissa Ilardi, Nikolay Pozdnyakov, Thomas A. Lanz, Hualin Xi, Robert D. Bell, Tarek A. Samad

**Affiliations:** 1 Pfizer Worldwide Research & Development, Cambridge, MA 02139 USA; 2 Present Address: Abbvie Inc., 200 Sidney St., Cambridge, MA 02139 USA; 3 Present Address: Biogen, 225 Binney St., Cambridge, MA 02142 USA; 4 Present Address: NYU School of Medicine, 550 1st Ave., New York, NY 10016 USA; 5 Present Address: Sanofi R&D, 49 New York Ave., Framingham, MA 01701 USA

**Keywords:** Monoacylglycerol lipase, 2-arachidonoylglycerol, Neuroinflammation, Blood-brain barrier, Neurovasculature

## Abstract

**Background:**

Acute neurological insults caused by infection, systemic inflammation, ischemia, or traumatic injury are often associated with breakdown of the blood-brain barrier (BBB) followed by infiltration of peripheral immune cells, cytotoxic proteins, and water. BBB breakdown and extravasation of these peripheral components into the brain parenchyma result in inflammation, oxidative stress, edema, excitotoxicity, and neurodegeneration. These downstream consequences of BBB dysfunction can drive pathophysiological processes and play a substantial role in the morbidity and mortality of acute and chronic neurological insults, and contribute to long-term sequelae. Preserving or rescuing BBB integrity and homeostasis therefore represents a translational research area of high therapeutic potential.

**Methods:**

Induction of general and localized BBB disruption in mice was carried out using systemic administration of LPS and focal photothrombotic ischemic insult, respectively, in the presence and absence of the monoacylglycerol lipase (MAGL) inhibitor, CPD-4645. The effects of CPD-4645 treatment were assessed by gene expression analysis performed on neurovascular-enriched brain fractions, cytokine and inflammatory mediator measurement, and functional assessment of BBB permeability. The mechanism of action of CPD-4645 was studied pharmacologically using inverse agonists/antagonists of the cannabinoid receptors CB1 and CB2.

**Results:**

Here, we demonstrate that the neurovasculature exhibits a unique transcriptional signature following inflammatory insults, and pharmacological inhibition of MAGL using a newly characterized inhibitor rescues the transcriptional profile of brain vasculature and restores its functional homeostasis. This pronounced effect of MAGL inhibition on blood-brain barrier permeability is evident following both systemic inflammatory and localized ischemic insults. Mechanistically, the protective effects of the MAGL inhibitor are partially mediated by cannabinoid receptor signaling in the ischemic brain insult.

**Conclusions:**

Our results support considering MAGL inhibitors as potential therapeutics for BBB dysfunction and cerebral edema associated with inflammatory brain insults.

**Electronic supplementary material:**

The online version of this article (10.1186/s12974-018-1166-9) contains supplementary material, which is available to authorized users.

## Background

The blood-brain barrier (BBB) is a selectively permeable barrier that regulates protein, metabolite, and ion transport to and from the central nervous system (CNS). It is composed of endothelial cells connected by tight junctions, pericytes, and astrocyte end-feet [1]. Acute neurological insults caused by infection, ischemia, or traumatic insults are often associated with breakdown of the BBB followed by infiltration of circulating immune cells and extravasation of plasma components. BBB breakdown and extravasation of these peripheral components into the brain parenchyma result in inflammation, oxidative stress, edema, excitotoxicity, and neurodegeneration [2]. Each of these insults can drive pathophysiological processes and play a substantial role in the morbidity and mortality of acute and chronic neurological diseases. Preserving or rescuing BBB integrity and function is therefore a research area with high therapeutic potential.

Systemic inflammatory challenges such as lipopolysaccharide (LPS), an immunogenic component of Gram-negative bacteria, promote BBB dysfunction [3]. Several inflammatory pathways have been proposed to contribute to the disruptive effects of LPS on BBB. Central to these mechanisms is the activation of the cerebrovascular endothelium and surrounding cells within the neurovascular unit by pro-inflammatory cytokines (IL-1β and TNFα) and eicosanoids induced by LPS [4].

Although the triggering mechanism of BBB disruption in ischemic stroke is relatively distinct from other systemic or central inflammatory stimuli [5], the neurovascular unit is dramatically sensitized to further disruptive changes by peripheral or central inflammation [6]. As a consequence, brain damage and mortality are exacerbated by systemic and central inflammation in clinical outcome as well as in experimental animal models [6, 7].

The interplay between brain injury and the subsequent inflammatory cascade as it pertains to BBB integrity and properties is not completely understood. Moreover, it is still unclear whether cerebrovascular changes and BBB breakdown can initiate pathogenic events that lead directly to neuronal injury, impaired functional activity, and early neurological symptoms in humans [2]. Therefore, a better mechanistic understanding of the molecular events leading to BBB dysfunction is necessary to address the unmet medical needs in acute brain injury and perhaps other chronic neurodegenerative conditions.

We and others have recently shown that monoacylglycerol lipase (MAGL), a serine hydrolase which modulates levels of the abundant endocannabinoid 2-arachidonoylglycerol (2-AG) [8, 9], also regulates neuroinflammation [10, 11]. Through hydrolysis of 2-AG, MAGL produces a pool of arachidonic acid (AA) which contributes to the inflammatory cascade in the CNS. Inhibition of MAGL enhances 2-AG-mediated cannabinoid receptor signaling while lowering arachidonate levels in the brain.

Interestingly, both pathways triggered by MAGL inhibition, elevation of 2-AG and reduction of AA, have been proposed to be implicated in modulation of BBB properties. After neurological insults, endocannabinoids are elevated and are hypothesized to protect the CNS through enhanced cannabinoid receptor signaling [12]. Indeed, administration of 2-AG was shown to be neuroprotective and restored BBB function after traumatic brain injury (TBI) [13]. In contrast, elevation of AA has long been known to induce BBB dysfunction and cerebral edema [14]. Due to the bidirectional effects on brain inflammation, MAGL inhibition has demonstrated a variety of beneficial therapeutic effects including improvement of BBB function in a TBI model [15]. However, the specific mechanism(s) of action of MAGL in BBB function and dysfunction has yet to be elucidated.

In this study, we assessed the effects of systemic inflammation on BBB function and the contribution of the inflammatory cascade to BBB dysfunction subsequent to an ischemic insult. We have characterized and used a selective inhibitor of 2-AG hydrolysis to modulate the central arachidonate inflammatory cascade and to enhance endocannabinoid tone. Transcriptomic analysis coupled with functional measurement of BBB integrity demonstrated that MAGL inhibition promotes preservation of BBB integrity in both inflammatory and ischemic conditions. In addition, we characterized the contribution of central arachidonate lowering effects relative to cannabinoid receptor agonism and demonstrated a dynamic and differential MAGL inhibition-mediated mechanism of BBB protection, which is dependent on the nature (inflammatory vs. ischemic) of the initial insult.

## Methods

### Animals

Animals were purchased from Charles River (CD1) or Jackson Labs (C57Bl/6). All animals were housed in groups in a temperature-controlled environment, kept on 12 h light/dark cycle, and allowed food and water ad libitum. All procedures were conducted under the approval of the Institutional Animals Care and Use Committee (IACUC) at Pfizer Inc. (Cambridge, MA).

### Pharmacokinetic, pharmacodynamic, cytokine, and activity-based protein profiling measurements

CPD-4645 was dissolved in a vehicle of 5:5:90 DMSO:Cremophor:Saline and subcutaneously administered to CD1 mice at a dose of 10 mg/kg. Plasma and brain samples of CPD-4645-treated mice were collected at 0.5, 1, 2, 4, 8, 12, and 24 h post-dose (three mice per time point). Plasma and brain samples of vehicle-treated mice were collected at 1 h post-dose.

Plasma samples were prepared for CPD-4645 measurement on wet ice. Briefly, samples were spiked with IS (internal standard) solution (100 ng/mL Labtalol, 400 ng/mL diclofenac, and 200 ng/mL tolbtamide in CAN/MeOH (*v*:*v* 50:50) with 0.1% formic acid) followed by extraction in water/MeOH (*v*:*v* 75:25) with 0.1% formic acid. Samples were then centrifuged at 4000 rcf for 10 min at 4 °C, and supernatant was directly injected for LC-MS/MS analysis. Cerebellums were homogenized with three volumes (*w*:*v*) of PBS. The homogenates were then spiked with IS solution followed by centrifugation at 15,700 rcf for 15 min at 4 °C. Supernatant was removed and directly injected for LC-MS/MS analysis.

Levels of brain 2-AG and AA were measured by homogenizing brain with three volumes (*w*:*v*) of homogenization buffer (1% PMSF and 5% 100 mM NH_4_OAc in water pH 2.0, adjusted with formic acid). The brain homogenates were then spiked with IS2 [1 μg/mL d_5_-2-AG or d_8_-AA (deuterated 2-arachidonoylglycerol or deuterated arachidonic acid) (Cayman Chemical Ann Arbor, Michigan) in ACN] solution followed by precipitation and organic extraction in ACN. Samples were then centrifuged at 15,700 rcf for 15 min at 4 °C and supernatant directly injected onto a C-18 UPLC column held at 50 °C at a flow rate of 0.5 mL/min. For 2-AG measurement, mass spectrometry was run in positive ESI mode with SRM detection. The 2-AG m/z was 379.4/287.3. The d_5_-2-AG m/z was 384.4/287.4. For AA measurement, mass spectrometry was run in negative ESI mode with SRM detection. The AA m/z was 303.1/205.2. The d_5_-2-AG m/z was 311.2/267.0. Calibration standards and quality control samples were prepared using the same methodology. The 2-AG calibration curve consisted of 50–50,000 ng/mL 2-AG in homogenization buffer. The AA calibration curve consisted of 50–50,000 ng/mL AA in homogenization buffer.

Brain cytokine levels were measured by preparing soluble proteomes as previously described [11] followed by ELISA measurement using V-Plex Proinflammatory Panel 1 (Meso Scale Diagnostics, Rockville MD) with detection antibodies for IL1β and IL6. Cytokine levels were normalized for total protein as determined by bicinchoninic acid (BCA) protein assay (Thermo Fisher, Waltham, MA).

Activity-based protein profiling was performed as previously described [9] with minor modifications. Briefly, membrane proteomes were isolated by homogenizing brain tissue in PBS buffer and centrifuged at 145,000×*g* for 45 min at 4 °C. Pellets were washed three times in PBS. Samples were diluted to 1 mg/mL total protein and incubated with 2 μM final fluorophosphonate-rhodamine. Reactions were incubated for 30 min at room temperature and quenched with 4× SDS loading buffer and boiled for 10 min at 95 °C. Samples were run on 12% SDS mini-gels and visualized using a fluorescent scanner (GE ImageQuant Las4000). Densitometry analysis was performed on the in-gel fluorescence images using Image Studio version 4 software (LI-COR, Lincoln, Nebraska).

### Induction of BBB disruption by lipopolysaccharide

Male CD1 mice aged 8–10 weeks were intraperitoneally injected with 3 mg/kg of salmonella enterica typhimurium (Sigma L2262) at 0, 6, and 24 h as previously described [3]. For the pharmacology studies, mice were dosed with 10 mg/kg subcutaneous CPD-4645 in a vehicle of 5:5:90 DMSO:Cremophor:Saline with and without combination of 3 mg/kg rimonabant and AM630 in vehicle (5:5:90; DMSO:Cremophor:Saline) 30 min post each LPS dose. For assessment of BBB function, mice were euthanized at 28 h after the first LPS injection. Animals were perfused with heparinized PBS, and brains were collected and frozen on dry ice for fluorescent immunostaining or ELISA. For the RNA-seq studies, the brains were not perfused as above and frozen brains were transferred into RNA-later-ICE Frozen Tissue Transition Solution (Life Technologies AM7030) in stored for 24 h at − 20 °C. Brain vasculature was then isolated as previously described [16] with the addition of two extra washes in sucrose buffer to remove remaining traces of myelin. Total RNA was purified using Qiagen RNeasy kits. RT-qPCR was performed to determine which cell types are present in the preparations.

**Table:** 

Gene symbol	Protein name	Taqman assay ID
Gfap	Glial Fibrillary Acidic Protein	Mm01253033_m1
Pdgfrb	Platelet-Derived Growth Factor Receptor Beta	Mm00435546_m1
Pecam1	Platelet Endothelial Cell Adhesion Molecule	Mm01242576_m1
Sypl	Synaptophysin-like	Mm01289818_g1
Aif1 (Iba1)	Allograft Inflammatory Factor 1	Mm00520165_m1

### Transcriptomics

#### Library preparation

Stranded cDNA libraries were prepared from 50 ng RNA using TruSeq Stranded mRNA NeoPrep kits (Illumina) and sequenced on a NextSeq 500 (Illumina) at a read depth of 10–20 million reads per sample (75 base pair single-end reads). FASTQ files were assembled using bcl2fastq.

#### Sequence data processing

Sequence reads were aligned to mouse genome mm10/GRCm38 assembly using STAR 2.5.2a (http://github.com/alexdobin/STAR; parameters: --outFilterMultimapNmax 20 --outFilterType BySJout --alignSJoverhangMin 8 --alignSJDBoverhangMin 1 --outFilterMismatchNmax 999 --outFilterMismatchNoverLmax 0.1 --alignIntronMin 20 --alignIntronMax 1000000 --alignMatesGapMax 1000000). Read counts for gene expression quantification were calculated using STAR --quantMode based on GENCODE release M9 basic annotation.

#### Differential gene expression analysis

The read count data were normalized by the trimmed mean of the *M*-values method [17] using the calcNormFactors() function from the edgeR package [18]. The mean-variance relationship of the counts was estimated using the voom() function [19] from the limma package [20]. To identify differentially expressed genes, the log_2_ fold differences and *p* values were estimated by fitting a linear model for each gene and applying empirical Bayes to moderate residual variances, using lmFit() and eBayes() functions from the limma package. Benjamini-Hochberg procedure for multiple hypothesis testing was applied to adjust *p* values. Differentially expressed genes were selected at twofold change (FC) cutoff and false discovery rate (FDR) of 0.05.

#### Gene ontology analysis

DAVID 6.7 bioinformatics tools (http://david-d.ncifcrf.gov) were applied for gene ontology (GO) analysis. The enriched GO categories were identified using the functional annotation clustering tool.

### Assessment of extravascular fibrinogen via fluorescent immunostaining

Fresh frozen brains were stored at − 20 °C until sectioning. Twelve-micrometer sections were cut using a cryostat and were heat mounted to slides and stored at − 20 °C until use. Sections were incubated in Zinc fix (BD Pharmingen #51-7538KZ) for 3 h prior to permeabilization (0.1% citrate, 0.1% Triton-X100 in PBS) for 10 min at 4 °C. Next, sections were blocked in 3% bovine serum albumin (BSA) for 1 h. Sections were incubated overnight at 4 °C in sheep anti-Fibrinogen (1:500; Serotec #4440-8004) and rat anti-CD31 (1:250; Serotec MCA2388T) made in 0.1% BSA. The next day, sections were washed in PBS-T and incubated in Alexa Fluor anti-sheep-555 (Invitrogen #A21436) and Alexa Fluor anti-rat-488 (Life Technologies #A21208) made in 0.1% BSA for 1 h at 37 °C. Slides were imaged on a Zeiss 710 confocal microscope. Images were taken with 20× objective and analyzed using ImageJ software. All images and subsequent analysis were performed blinded. To analyze extravascular fibrinogen, images were thresholded to remove signal where CD31 staining (vascular endothelial marker) and fibrinogen staining were co-localized. The areas of exclusively fibrinogen signal in the brain were quantified using the ImageJ integrity density function.

### Measurement of brain and plasma fibrinogen levels

Brain homogenates were made by sonicating one cerebral hemisphere (without cerebellum) in radioimmunoprecipitation assay buffer (Sigma #R0278) containing protease and phosphatase inhibitors (Pierce #88669). Lysates were centrifuged twice at 13,300 rpm for 15 min. Pellets were discarded and supernatants were kept for analysis. For plasma sample collection, blood samples were collected in heparin-sodium (1:10) via cardiac puncture and centrifuged at 6000 rpm for 5 min. Plasma was collected and spun again at 13,300 rpm for 5 min to remove any residual blood cells. Brain homogenates (1:30 dilution) and plasma samples (1:20,000 dilution) were analyzed for fibrinogen levels by ELISA (Genway; cat #GWBBB0BA2) following the manufacturer’s protocol.

### Measurement of FITC dextran permeability in LPS model

At 1 h prior to tissue collection, 200 μL of 25 mg/mL solution of fluorescein isothiocyanate (FITC) conjugated dextran (70 kDa, Sigma Aldrich, St Louis, MO) in sterile saline was injected retro-orbitally. At 1-h post-fluorescent tracer administration, mice were anesthetized with isoflurane and transcardially perfused with approximately 20 mL of phosphate buffered saline (PBS) with heparin (5 U/ml). The brain was collected, frozen in liquid nitrogen, and stored at − 80 °C until use.

To measure FITC-Dextran fluorescence, a hemi-brain was homogenized using a TissueLyser II (Qiagen, Germantown, MD) set at 25 Hz for 2 min at 4 °C in 600 μL of ice-cold radioimmunoprecipitation (RIPA) assay buffer (150 mM NaCl, 1.0% IGEPAL® CA-630, 0.5% sodium deoxycholate, 0.1% SDS, and 50 mM Tris, pH 8.0) (Sigma Aldrich, St Louis, MO) with protease inhibitors (cOmplete EDTA-free, Roche, Indianapolis, IN). Each sample was then centrifuged at 10,000 rcf for 10 min at 4 °C to pellet large debris.

Supernatant was collected and diluted 1:1 with PBS placed into a black 96-well plate. Fluorescence was measured in a SpectraMax M5 (Molecular Devices, Sunnyvale, CA) with excitation at 488 nm and emission at 525 nm. After the initial reading, samples were further diluted (1:4 and 1:8) and fluorescence intensity re-measured. Each reading was multiplied by the corresponding dilution factor, and an average fluorescence reading was determined.

### Induction of focal photothrombotic ischemia

To alleviate any potential pain or discomfort, mice were treated with 0.05 mg/kg of buprenorphine prophylactically. Mice were anesthetized with isoflurane for the duration of the procedure. Mice (C57BL/6) received a retro-orbital injection of 30 μL of a 15 mg/mL solution of rose bengal dissolved in sterile saline. The head of the mice was shaved and a small incision aseptically made along the midline of the skull. The mice were then placed into a stereotaxic frame, and a green laser held approximately 4 cm from the surface of the skull was positioned over the somatosensory cortex. Mice were subjected to the laser exposure for 10 min. After green laser illumination, the skin incision was closed with wound clips and animals monitored as they woke from the anesthesia. Mice were subcutaneously administered vehicle or 10 mg/kg CPD-4645 30 min post-photothrombosis. Mice received a daily injection of vehicle of CPD-4645 for 3 days.

On day 3, each mouse was retro-orbitally injected with Alexa Fluor® 555-conjugated Cadaverine (Thermo A30677) at 500 μg/20 g body weight as previously described [21] or 200 μL of 25 mg/mL solution of fluorescein isothiocyanate (FITC)- conjugated dextran (70 kDa, Sigma Aldrich, St Louis, MO) in sterile saline. At 1 h post-fluorescent tracer administration, mice were anesthetized with isoflurane and transcardially perfused with approximately 20 mL of PBS with heparin (5 U/ml). The brain was collected and the cortex dissected. A 5-mm tissue punch was taken from the core of the thrombotic infarct and from the identical region of contralateral cortex. Tissues were frozen in liquid nitrogen and stored at − 80 °C until use.

To measure FITC-Dextran fluorescence, a 5-mm stainless steel bead was added to each tube along with 300 μL of ice-cold radioimmunoprecipitation (RIPA) assay buffer (150 mM NaCl, 1.0% IGEPAL® CA-630, 0.5% sodium deoxycholate, 0.1% SDS, and 50 mM Tris, pH 8.0) (Sigma Aldrich, St Louis, MO) with protease inhibitors (cOmplete EDTA-free, Roche, Indianapolis, IN). Samples were then homogenized using a TissueLyser II (Qiagen, Germantown, MD) set at 25 Hz for 2 min at 4 °C followed by incubation on ice for 30 min. Each sample was then centrifuged at 10,000 rcf for 10 min at 4 °C to pellet large debris.

Supernatant was collected and diluted 1:1 with PBS placed into a black 96-well plate. Fluorescence was measured in a SpectraMax M5 (Molecular Devices, Sunnyvale, CA) with excitation at 488 nm and emission at 525 nm. After the initial reading, samples were further diluted (1:4 and 1:8) and fluorescence intensity re-measured. Each reading was multiplied by the corresponding dilution factor, and an average fluorescence reading was determined.

## Results

### Profiling of inflammatory gene expression in neurovascular-enriched brain fraction following systemic insult

**Fig. 1:**
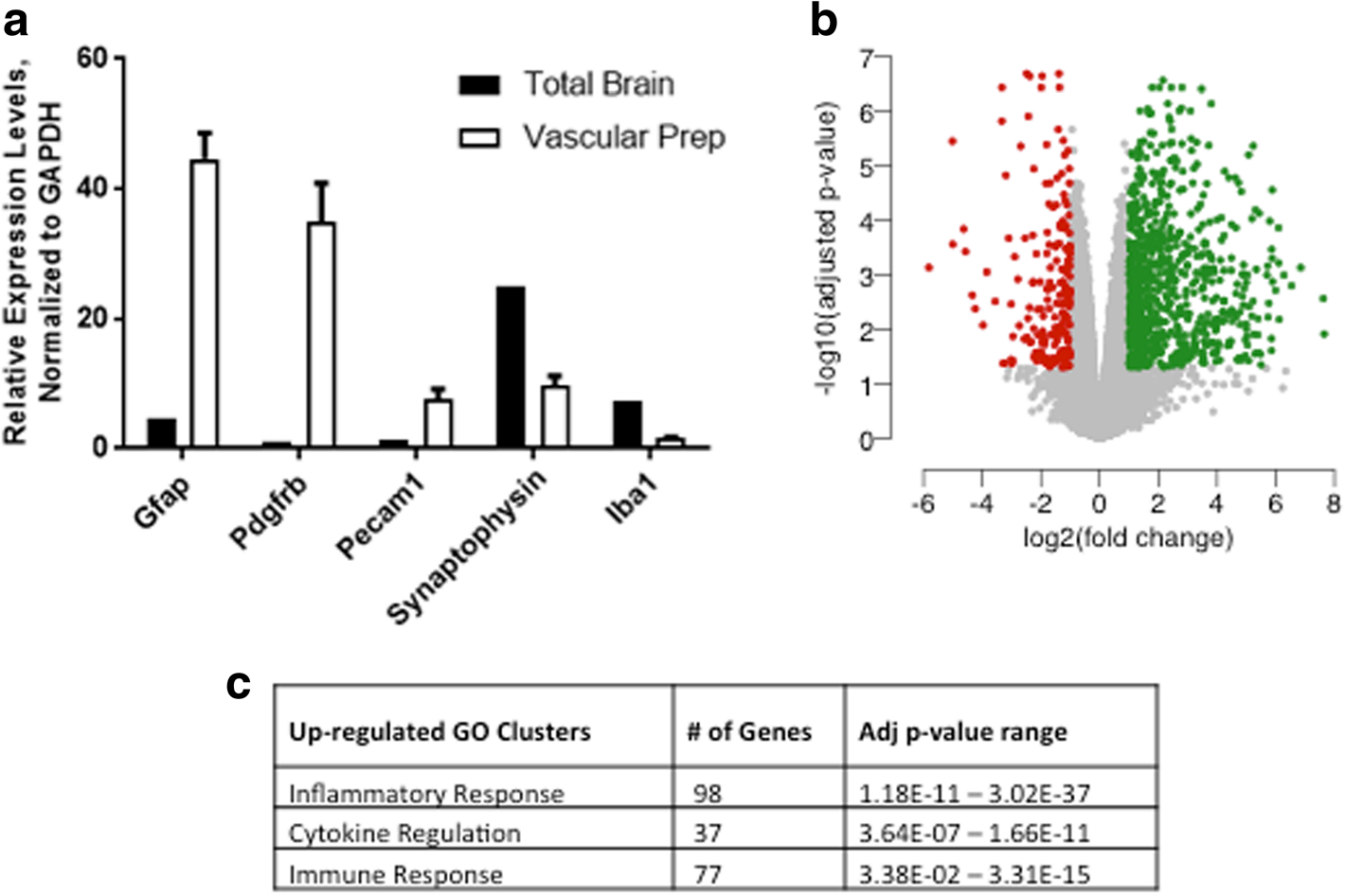
Transcriptomic profiling of neurovascular unit after LPS challenge. **a** Expression profile of cell type-specific makers in neurovasculature preparation. **b** Volcano plot of statistically significant upregulated (green) and downregulated (red) genes in the neurovascular unit after LPS challenge. **c** Gene ontology clusters for up- and downregulated genes identified using DAVID bioinformatics resource. Neurovasculature was isolated 4 h post last LPS dose. Data are means ± SEM, *n* = 4/5 mice per group

Brain fraction enriched for cerebrovascular tissue was isolated as previously described [16]. To confirm enrichment of the neurovasculature, we assessed expression of cell type-specific genes characteristic of the different cell types that compose the blood-brain barrier. Gene expression analysis by RT-qPCR demonstrated enrichment in the expression of *Gfap*, *Pdgfrb*, and *Pecam1* and low abundance of *Syp* and *Aif1* (Fig. 1a) demonstrating prevalence of astrocytes, pericytes, and endothelial cells over neurons and microglia which is consistent with the cell types that comprise the neurovascular unit.

Activation of the neurovasculature through inflammatory challenge has previously been shown to disrupt the integrity of the BBB and substantially induce its permeability [3, 22]. After systemic LPS administration, brain vasculature was isolated and extracted RNA was analyzed by next generation sequencing for gene expression quantification. The RNA-seq analysis identified 949 differentially regulated genes (false discovery rate ≤ 0.05, increased or decreased by at least twofold) by LPS challenge compared to vehicle controls (Fig. 1b and Additional file 1: Table S1). Among those, 745 were statistically significantly upregulated, and 204 were significantly downregulated. The GO analysis of the upregulated genes using DAVID bioinformatics tools identified the top three enriched functional annotation clusters as inflammatory response, regulation of cytokine production, and immune response (Fig. 1c and Additional file 2: Table S2). The inflammatory response cluster contained genes pertaining to TLR (Toll-like receptor) signaling and inflammasome activation as expected in an LPS challenge model. The cytokine regulation and immune response clusters contained a number of genes coding for cytokines, chemokines, and complement system proteins. Of the LPS downregulated genes, there were no statistically significant clusters identified (Additional file 2: Table S2), and therefore in this study, we only focus on the upregulated genes.

### Inhibition of 2-AG hydrolysis rescues altered transcriptional profile of the neurovascular unit after LPS challenge

Previous studies have highlighted the anti-inflammatory effects of MAGL inhibition in acute and chronic brain insults and transgenic mouse models [10, 11, 23, 24]. Additionally, the protective role of 2-AG in preserving BBB integrity after acute brain injury has been described [13]. Therefore, we sought to determine whether inhibition of MAGL activity in vivo can reverse BBB pathogenesis and rescue neurovascular integrity and function after insults.

**Fig. 2:**
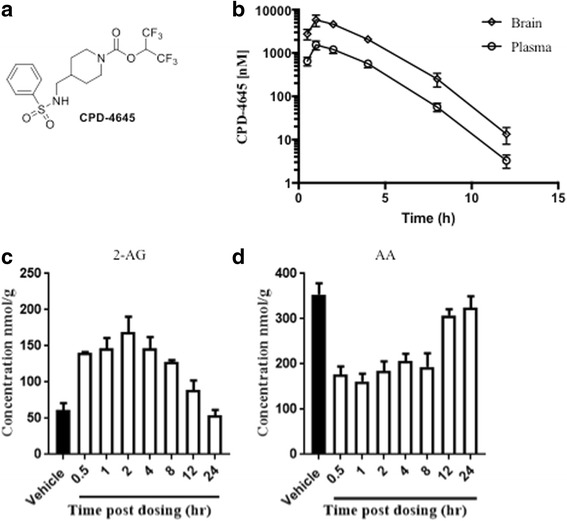
Pharmacokinetic and pharmacodynamic profiling of CPD-4645 in naïve mouse brain. **a** Structure of the 2-AG hydrolysis inhibitor CPD-4645. Total CPD-4645 concentrations in brain and plasma (**b**) and bulk levels of brain 2-AG (**c**) and AA (**d**) at given time points following single 10 mg/kg subcutaneous dose of CPD-4645 in CD1 mice. Data are means ± SEM, *n* = 3/5 mice per group

CPD-4645 (Fig. 2a) is a hexafluoroisopropyl carbamate covalent MAGL inhibitor [25]. CPD-4645 is brain penetrant (Fig. 2b), and administration of subcutaneous CPD-4645 at 10 mg/kg to CD1 mice resulted in ~ 3-fold increase in brain 2-AG (Fig. 2c) which persisted for 8 h. Levels of brain AA were reduced by 50% over the same time period (Fig. 2d). CPD-4645 inhibited MAGL (~ 99% max), alpha-beta-hydrolase domain containing 6 (ABHD6) (~ 70% max), and fatty acid amide hydrolase (FAAH) (~ 50% max) in the brain as determined by activity-based protein profiling (Additional file 3: Figure S1). No inhibition of additional serine hydrolases was detected (data not shown). ABHD6 and FAAH are serine hydrolases responsible for the degradation of 2-AG and anandamide (AEA), respectively. ABHD6 is closely related to MAGL and is responsible for ~ 15% of the hydrolysis of 2-AG while MAGL is responsible for the remaining ~ 85% [26]. Although the majority of the pharmacodynamic effects seen with CPD-4645 may be attributed to inhibition of MAGL, the partial inhibition of ABHD6 may also be contributing to the 2-AG elevation and AA lowering seen in the brain. It has previously been shown that > 85% inhibition of FAAH is required to maintain significant elevations in brain AEA levels [27], as such the 50% inhibition of FAAH seen in these experiments is below the threshold level needed to elicit a pharmacodynamic effect.

**Fig. 3:**
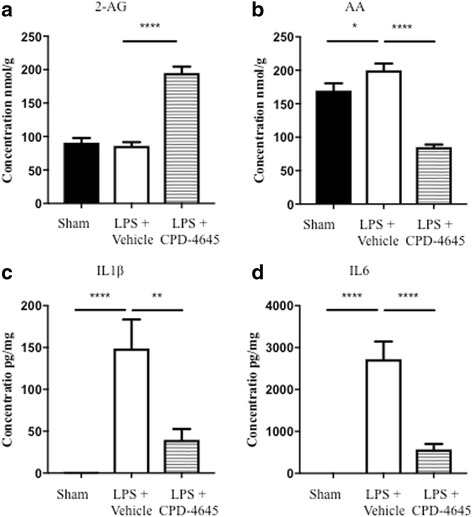
Pharmacodynamic and anti-inflammatory activity of CPD-4645 in LPS-challenged mouse brain. Inhibition of MAGL by CPD-4645 resulted in significant elevation of brain 2-AG (**a**) and concomitant reduction in brain AA (**b**). Levels of the proinflammatory cytokines, IL1β (**c**) and IL6 (**d**), were significantly modulated in brain tissue following LPS challenge and CPD-4645 treatment. Bar graphs were plotted with mean ± SEM and data analyzed using one-way analysis of variance (ANOVA) with Bonferroni post-hoc comparisons. *n* = 10 mice per group. Significance is shown as *p < 0.05; **p < 0.01; ****p < 0.0001

**Fig. 4:**
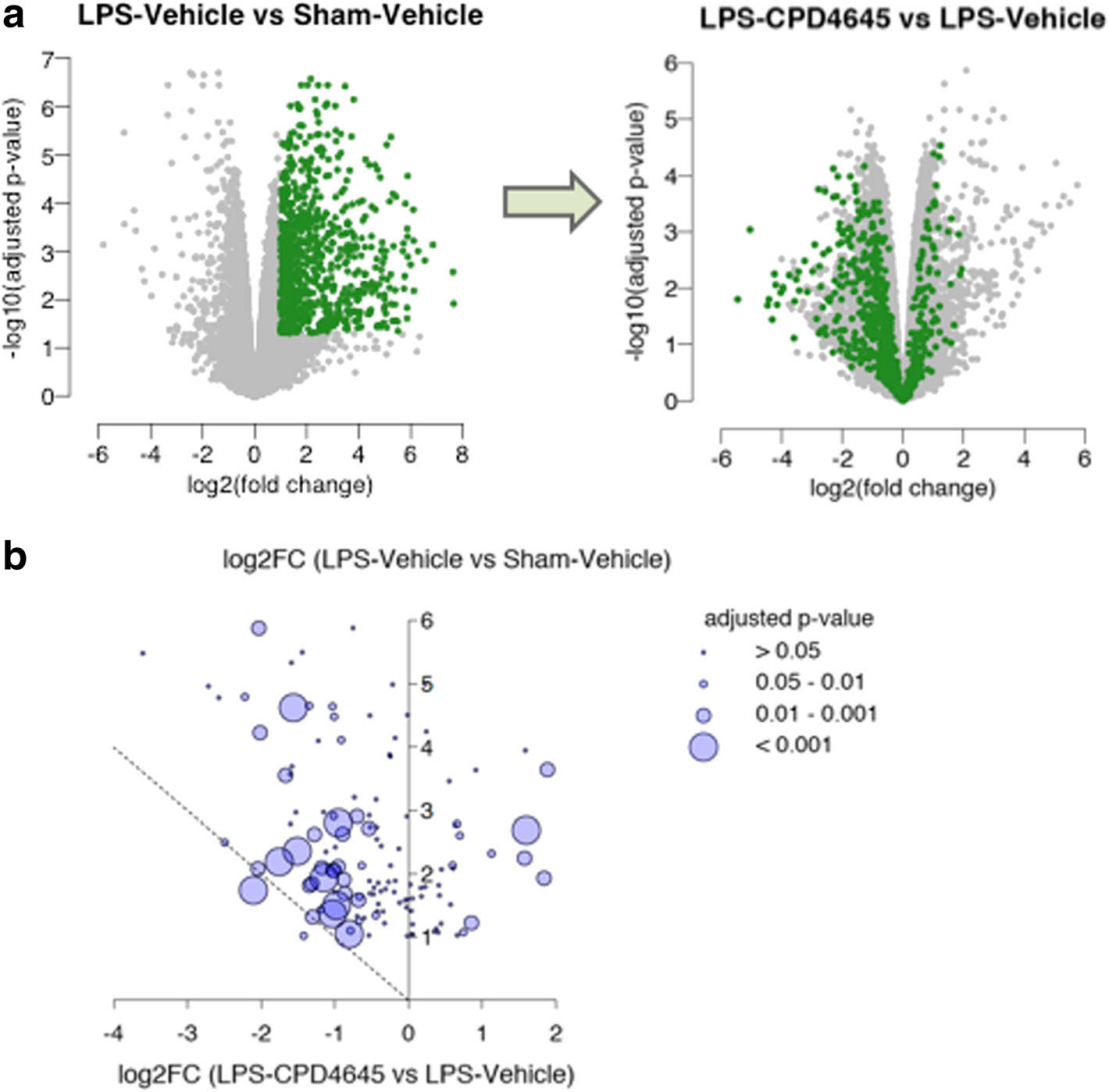
CPD-4645 alters LPS-induced gene expression profiles. **a** Volcano plots showing expression changes of the LPS upregulated genes (left, green points) after treatment with CPD-4645 (right). **b** Bubble plot showing changes in expression of genes that are induced by LPS and related to cytokines and inflammation. The *y*-axis represents the log2-fold change due to LPS challenge while the *x*-axis represents the log2-fold change due to CPD-4645 treatment. Dotted line represents a return to basal expression level. Size of the bubbles represents the multiple comparison adjusted *p* values for vehicle versus CPD-4645 treatment. Neurovasculature was isolated 4 h post last LPS dose. *n* = 4/5 mice per group

Mice were subjected to the LPS challenge as described above, in the presence and absence of CPD-4645 which was administered 45 min post each dose of LPS. Assessment of brain 2-AG levels 3.5 h post last dose of CPD-4645 revealed no effect of LPS while CPD-4645 treatment significantly elevated levels of the endocannabinoid (Fig. 3a). There was a modest but significant elevation in brain AA levels due to LPS challenge, which was significantly attenuated by CPD-4645 treatment (Fig. 3b). Given the potent anti-inflammatory effects of MAGL inhibition, we next measured levels of the proinflammatory cytokines IL1β and IL6 in the brains of mice challenged with LPS. The LPS challenge significantly elevated levels of both cytokines above baseline levels while treatment with CPD-4645 significantly reduced the inflammatory mediators (Fig. 3c, d). We next profiled the effects of LPS and CPD-4645 on the transcriptional profile of the neurovasculature. As expected, transcriptional signatures showed a clear distinction between the sham and LPS group (separated by the first principal component as shown in Additional file 4: Figure S2). However, the CPD-4645 treatment exhibited a striking reversal of the transcriptional induction observed in the LPS group (Fig. 4a). Among the 745 statistically significantly upregulated genes in response to LPS challenge, the expression of 550 (74%) showed reduction in gene expression with CPD-4645 treatment (95% confidence interval 0.70–0.77, proportion test). The majority of the LPS upregulated genes in the annotation clusters related to inflammation, cytokine regulation, and immune response (Fig. 1c and Additional file 2: Table S2) were attenuated by CPD-4645 treatment (Fig. 4b). The top enriched GO clusters identified for the significantly downregulated genes (Additional file 1: Table S1) in the CPD-4645-treated animals included leukocyte migration, inflammatory response, and chemotaxis (Additional file 2: Table S2). These results suggest a role of MAGL inhibition in reducing mediators of leukocyte-endothelial cell interaction and inflammation.

**Fig. 5:**
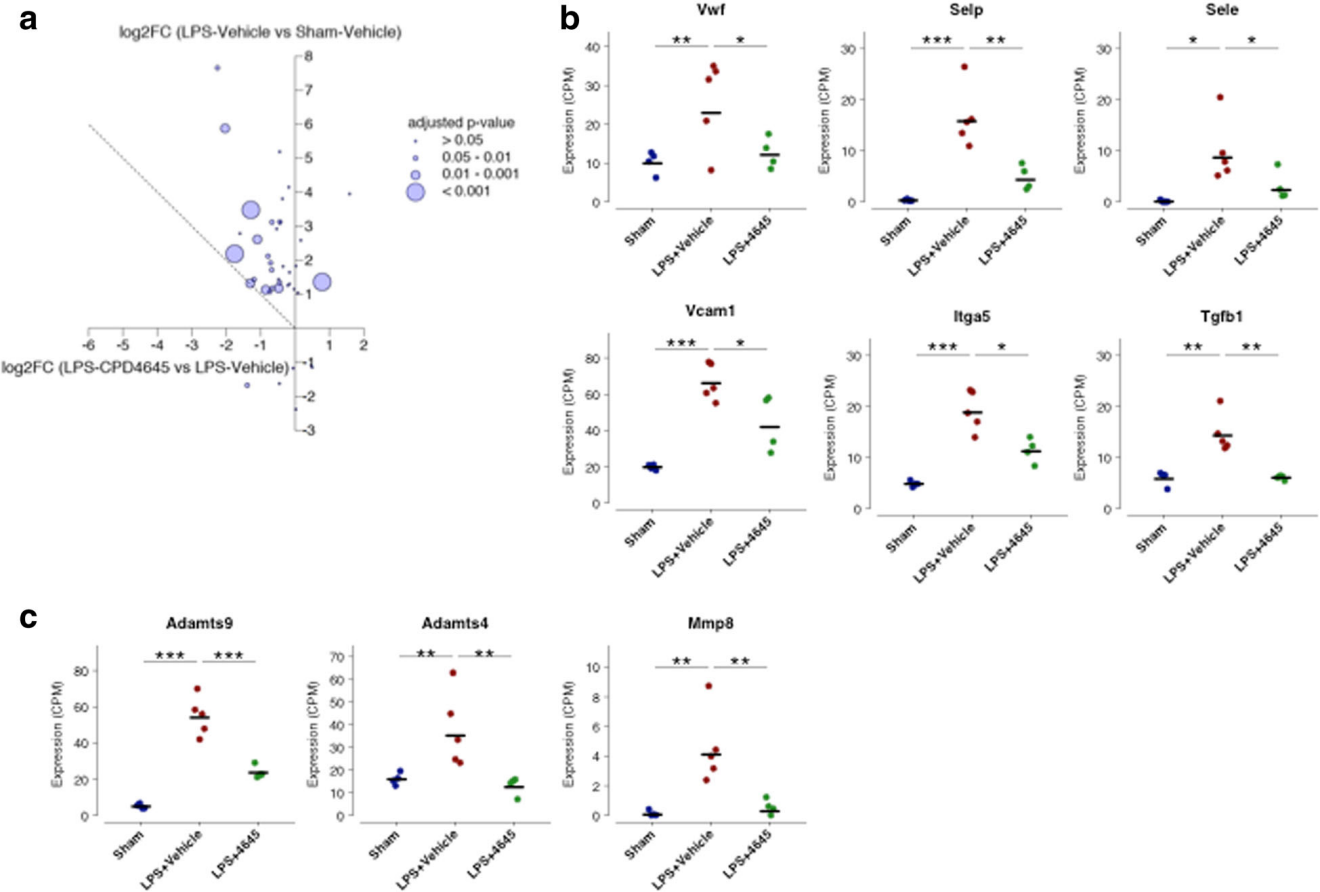
Genes related to BBB (dys) function and proteases are differentially expressed after LPS challenge and treatment with CPD-4645. **a** Changes in the expression of 40 genes related to BBB function and dysfunction. The *y*-axis represents the log2-fold change due to LPS challenge while the *x*-axis represents the log2-fold change due to CPD-4645 treatment. Dotted line represents a return to basal expression level. Size of the bubbles represents the multiple comparison adjusted *p* values for vehicle vs. CPD-4645 treatment. **b** Expression levels of selected BBB genes. **c** Expression levels of selected extracellular proteases. *n* = 4–5 mice/group. Significance is shown as adjusted *p* values *p ≤ 0.05, **p < 0.01, *** *p* < 0.001. Differential gene expression was analyzed using the limma package in R/Bioconductor (see Methods)

In addition to the notable changes in genes directly involved in immune and inflammatory responses, several genes associated with endothelial activation and loss of BBB integrity were significantly altered by LPS challenge and attenuated in animals treated with CPD-4656 (Fig. 5a). We found that in mice treated with CPD-4645, the transcript levels of *Vwf*, *Selp*, *Sele*, *Vcam1*, *Itga5*, and *Tgfb1*, markers of endothelial cell activation and inflammatory responses, were significantly reduced compared to vehicle-treated animals following the LPS challenge (Fig. 5b). In addition, transcript levels of the extracellular proteases *Adamts9*, *Adamts4*, and *Mmp8* were also upregulated by LPS challenge and significantly reduced following CPD-4645 treatment (Fig. 5c).

### Inhibition of 2-AG hydrolysis reduces BBB permeability after LPS challenge

**Fig. 6:**
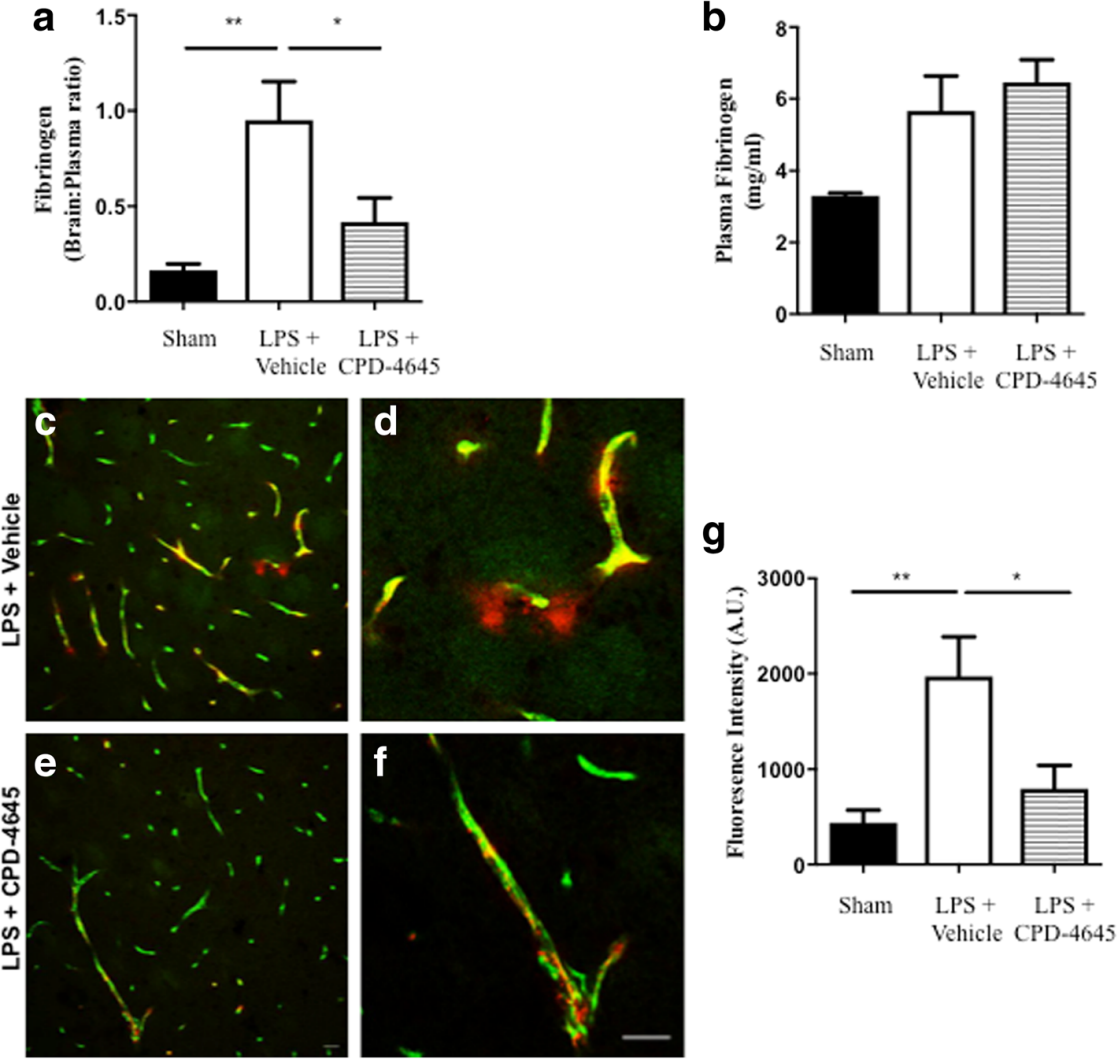
Inhibition of 2-AG hydrolysis reduces LPS-induced BBB permeability. **a**, **b** Fibrinogen levels in **b** plasma and the **a** ratio of brain to plasma fibrinogen were assessed by ELISA. *n* = 5/7 mice per group. **c**, **d** Fluorescent immunostaining in the striatum for fibrinogen (red) and vascular marker (CD31; green) demonstrated leakage of fibrinogen into the brain with vehicle treatment, whereas vascular integrity was preserved when (**e**, **f**) MAGL was inhibited. **g** Extravascular fibrinogen was semi-quantitated in fluorescently labeled sections of the striatum. Bar graphs were plotted with mean ± SEM and data analyzed using one-way analysis of variance (ANOVA) with Tukey post-hoc comparisons. *n* = 5/7 mice per group. Significance is shown as *p < 0.05, **p < 0.01. Scale bar = 20 μm

In order to assess the physiological consequences of reversing the inflammatory signature of brain endothelial activation and dysfunction, we evaluated BBB integrity by measuring extravasation of an abundant plasma protein, fibrinogen, into the brain via ELISA analysis and immunofluorescent staining in LPS-challenged animals treated with either vehicle or CPD-4546. We measured the ratio of the brain to plasma fibrinogen using ELISA. As fibrinogen is an acute phase protein upregulated under inflammatory conditions, to account for potential differences in plasma fibrinogen, we normalized the brain fibrinogen to the amount of fibrinogen present in the plasma. We showed that the ratio of the brain to plasma fibrinogen was significantly decreased in animals treated with CPD-4645 following LPS challenge (Fig. 6a). Of note, LPS challenge resulted in elevated levels of circulating fibrinogen in both vehicle- and MAGL inhibitor-treated mice to the same degree, suggesting that reduction of brain fibrinogen in the LPS/CPD-4645 group relative to LPS/veh is not due to reduction in systemic fibrinogen (Fig. 6b). In addition, we stained for CD31+ endothelial cells and fibrinogen in the striatum. Images were merged to determine the degree of extravascular fibrinogen staining as previously reported [28]. Vehicle-treated mice show significant leakage of fibrinogen into the brain parenchyma (Fig. 6c, d, and g) a phenotype that was significantly reversed when LPS-challenged animals were treated with CPD-4546 (Fig. 6e–g).

### Inhibition of 2-AG hydrolysis reduces blood-brain barrier permeability following photothrombosis

**Fig. 7:**
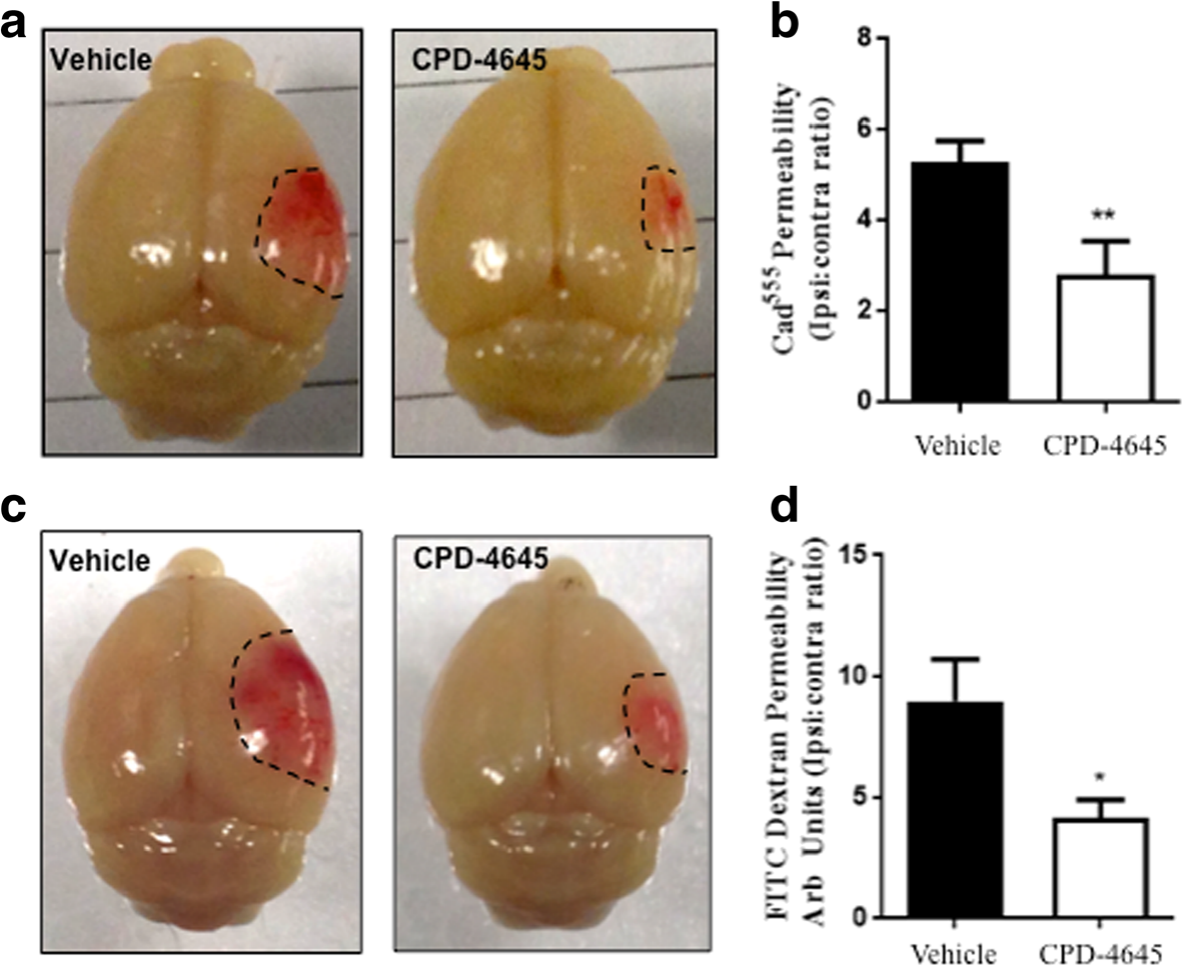
CPD-4645 rescues BBB integrity after ischemic challenge. The rose bengal photothrombosis model induces a focal ischemic injury that leads to BBB dysfunction. CPD-4645 administered 30 min post lesion (**a**, **b**) reduced the penumbra size (**a**) and BBB permeability (**b**) as assessed by extravasation of Cadavarin^555^ into the brain parenchyma. CPD-4645 administered 6 h post ischemic lesion (**c**, **d**) reduces penumbra size (**c**) and BBB permeability (**d**) as assessed by extravasation of 70 kDa FITC conjugated dextran in the brain parenchyma. Data were plotted with means ± SEM and data analyzed using unpaired *t* test (*n* = 5 mice/group). Significance is shown as *p ≤ 0.05 and **p < 0.01 compared to vehicle group

Ischemic injury results in a brain inflammatory cascade that also alters BBB properties and permeability. To assess efficacy of MAGL inhibition at reducing BBB permeability following ischemic inflammatory injury, the photosensitizing dye, rose bengal, was combined with exposure to green laser light to induce focal ischemic injury in the somatosensory cortex which results in BBB breakdown [29, 30]. The extravasation of high molecular weight fluorescent tracers, which are typically excluded from the brain parenchyma, was utilized as a surrogate marker of altered BBB permeability. Treatment with CPD-4645 30 min after inducing the lesion resulted in significant attenuation of BBB damage (Fig. 7a, b). To further test the clinical utility of a MAGL inhibitor, animals were treated 6 h after the onset of the ischemic lesion. Interestingly, leakage of the fluorescent tracer was significantly attenuated in CPD-4645-treated animals compared to vehicle (Fig. 7c, d). These results indicate that inhibition of MAGL, even when withheld for 6 h, could significantly improve BBB function after ischemic injury.

### Differential mechanisms of action of 2-AG hydrolysis inhibition in models of BBB damage

**Fig. 8:**
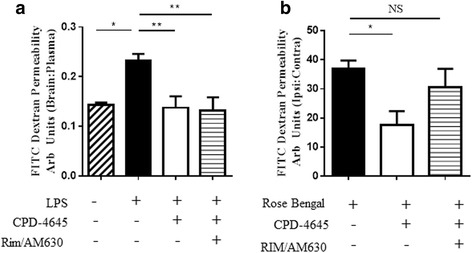
CPD-4645 rescues BBB integrity via endocannabinoid dependent and independent mechanisms. Blockade of endocannabinoid signaling with rimonabant and AM630 (3 mg/kg) does not reverse the BBB protective effects of MAGL inhibition in the inflammation-driven LPS model (**a**), whereas blockade of endocannabinoid signaling partially reverses the protective effects of MAGL inhibition (administered 30 min post lesion) in the photothrombotic ischemia-driven model (**b**) as measured by extravasation of 70 kDa FITC-dextran into the brain parenchyma. Data were plotted with means ± SEM (*n* = 6–9 mice/group) and analyzed with one-way ANOVA with Bonferroni’s post-hoc test. Significance is shown as *p ≤ 0.05, **p ≤ 0.01

To assess the contribution of cannabinoid signaling to the mechanism of action of MAGL inhibition in preservation of blood-brain barrier integrity, we tested if the efficacy of MAGL (and possibly ABHD6) inhibition was reversed by blockade of cannabinoid type 1 and 2 receptor signaling. A combination of CB1 and CB2 receptor antagonists, Rimonabant and AM630 (3 mg/kg s.c.), were used in the LPS- and focal ischemia-induced BBB disruption models. Co-treatment of LPS-challenged mice with CB1 and CB2 antagonists did not significantly reverse the effect of MAGL inhibition on BBB breakdown (Fig. 8a). In contrast, co-treatment of photothrombotic mice with MAGL inhibitor and CB1 and CB2 antagonists partially reversed the protective effect of MAGL inhibition on BBB dysfunction (Fig. 8b). These results suggest that 2-AG hydrolysis differentially regulates BBB permeability after systemic or ischemic inflammatory injury, and that enhanced cannabinoid signaling, through CB1 and/or CB2, contributes to the rescue of BBB permeability following ischemic injury. The contribution of cannabinoid signaling to BBB rescue in the LPS model still cannot be ruled out based on these results alone and will require additional molecular and pharmacological characterizations.

## Discussion

The integrity of the BBB is often compromised following acute insults or in cases of chronic neuroinflammation leading to infiltration of circulating immune cells and extravasation of blood components into the brain parenchyma. The entrance of these restricted components into the CNS can lead to a multitude of pathophysiological responses and further exacerbate the initial trauma or disease. In this study, we demonstrate that inhibition of 2-AG hydrolysis (inhibition of MAGL and partial inhibition of ABHD6) by CPD-4645 is sufficient to reduce BBB damage in both inflammatory- and ischemic-driven models of BBB dysfunction.

We show that CPD-4645 is a potent and brain penetrant covalent MAGL inhibitor. Treatment with CPD-4645 was sufficient to elevate brain levels of the major endocannabinoid, 2-AG, while reducing levels of brain AA in naïve and LPS-challenged mice. In addition to modulating AA levels, inhibition of 2-AG hydroylsis resulted in significant attenuation of the proinflammatory cytokines IL1β and IL6 in the brains of LPS-challenged mice. These results highlight the potent anti-inflammatory mechanism of MAGL inhibition.

RNA-seq analysis of isolated brain vasculature after LPS challenge showed a significant elevation of genes related to the immune and inflammatory responses as well as endothelial cell activation. Treatment with CPD-4645 was able to restore these gene clusters closer to sham-treated levels. The results of this particular experiment are confounded by the fact that peripheral immune cells like macrophage and neutrophils may be attaching to the cerebral endothelium and/or entering the brain after LPS challenge. The transcriptomic signature shown in this study is a combination of all cells present in the isolated neurovascular unit preparation including those peripheral cells. The changes in gene expression are therefore a snapshot of the overall state of health of the BBB and do not necessarily imply that MAGL inhibition is directly responsible for altering transcription.

Further analysis into the gene expression dataset revealed a number of interesting findings related to endothelial activation, leukocyte-endothelial interaction, and BBB integrity-related genes. Although assumed based on previous reports, our RNA-seq results confirmed that endothelial activation occurs in this LPS model as is evidenced by the upregulation of von Willebrand factor (*Vwf*), P-Selectin (*Selp*), and E-Selectin (*Sele*). *Vwf* and *Selp* are two key components of Weibel-Palade bodies which are released from endothelial cells in response to cytokines, playing dual roles in regulating hemostasis and additional inflammatory processes [31]. Interestingly, *Selp* knockout mice show reduced BBB breakdown following transient ischemic stroke, likely due to preventing leukocyte-endothelial cell interaction and downstream inflammatory process damaging the endothelial cell integrity [32]. In addition, mice genetically engineered to produce high levels of soluble *Selp* in circulation showed an increase in BBB permeability [33]. We also demonstrated CPD4-656 treatment reduced the upregulation of vascular cell adhesion molecule 1 (*Vcam1*), which is known to occur in response to cytokine release and plays an additional roll in leukocyte-endothelial interactions. Similar to P-selectin, soluble Vcam1 in CSF and plasma correlates with BBB lesion severity, in multiple sclerosis (MS) for example [34]. Moreover, the mechanism of action of the approved MS therapy Natalizumab involves prevention of the α4β1-integrin receptor molecules on immune cells from interacting with Vcam1 on endothelial cells to reduce disease-related lesions and leukocyte trafficking into the brain.

The interaction of endothelial cells with the extracellular matrix protein fibrinonectin is mediated through integrin alpha-5 (*Itga5*). It was recently found that endothelial Itga5 protein levels are increased in the inflammatory EAE model of multiple sclerosis and coincide with fibrinogen leakage into the spinal cord [35]; thus, our findings that *Itga5* mRNA is upregulated with LPS treatment is not surprising. Moreover, deletion of *Itga5* specifically in endothelial cells strengthens BBB integrity and increases tight junction protein expression following acute brain injury [36]. Interestingly, MAGL inhibition following LPS challenge also reduced the levels of *Tgfb1*, a known upstream regulator of *Itga5* and other integrins [37]. The action of *Tgfb1* on the vasculature is complex and highly dependent on the cellular and environmental context [38]. Whether the effects of CPD-4645 on *Itga5* expression are dependent or independent on *Tgfb1* signaling remains elusive and should be addressed in future experiments.

Although interesting, it is currently unknown whether the attenuation of upregulation of these genes in LPS-challenged animals with CPD-4645 treatment is a direct result of inhibiting the MAGL pathway in endothelial cells or is due to an indirect effect of reducing neuroinflammation. Regardless, maintenance of these genes at a baseline level after exposure to LPS most likely plays a role in preservation of BBB as these proteins have been implicated in modulation of BBB integrity [5, 39].

Gene expression analysis also revealed changes in the matrix metalloproteinases (MMPs) and the disintegrin and metalloproteinases with thrombospondin motifs (ADAMTSs). MMPs and ADAMTs are classes of extracellular proteases which cleave a wide variety of extracellular matrix, tight junction, and basement membrane proteins leading to the hypothesis that their activities contribute to the dysfunction of the BBB in inflammatory and ischemic injuries [40–43]. In this study, we found LPS challenge to induce the expression of several MMPs including *Mmp-3*, *8*, *9*, and *19*. Of these, *Mmp8* was significantly reduced by treatment with CPD-4645. MMP-8 is an inducible MMP that has been shown to be upregulated in experimental models of multiple sclerosis [44] and elevated in the CSF of children with bacterial meningitis [45]. Consistent with our findings in the LPS model, which mimics a bacterial challenge, infection of human brain microvascular endothelial cells with *Neisseria meningitides* resulted in an increase in MMP-8 activity and was shown to be directly involved in the cleavage of the occludin and subsequent BBB dysfunction [46].

The role of ADAMTS family of proteins has been extensively studied for their ability to remodel extracellular matrix (ECM) in the context of osteoarthritis. Recent emerging data suggest that astrocytic ADAMTSs may play a variety of roles within the CNS including modulation of neuronal signaling, inflammation, and permeability of the BBB [42]. We found that LPS challenge stimulated the expression of *Adamts-1*, *4*, *9*, and *14* in the neurovasculature. Treatment with CPD-4645 significantly downregulated expression of *Adamts4* and *9*. It has been shown that IL1β and TNFα, cytokines which are elevated in our LPS model, can induce the expression of *Adamts4* and *9* [47, 48] in astrocytes and macrophage. Versican is the major chondroitin sulfate proteoglycan within the vasculature and is a cleavage substrate for ADAMTS-4 [49] and ADAMTS-9 [50] leading to the hypothesis that ADAMTS activity may contribute to BBB dysfunction via degradation of ECM [42, 51].

Taken together, our data suggest that inflammation induced by LPS challenge can serve to upregulate expression of *Mmp8* and *Adamts4* and *9* which may contribute to subsequent damage of the BBB through degradation of occludin and versican. Treatment with a MAGL inhibitor elicits an anti-inflammatory response which in turn serves to reduce levels of the neurovascular damaging proteases. Regulation of proteases through reduction in inflammation is one possible explanation of many for the BBB protective effects observed in this study. A deeper understanding of the complex interplay between neuroinflammation, protease activity, and BBB function will be required to determine if MMP-8, ADAMTS-4, or ADAMTS-9 are solely responsible or act in concert with each other or with other pathophysiological processes leading to BBB dysfunction.

Several studies have shown that augmentation of 2-AG, either through direct administration or through inhibition of MAGL or ABHD6, can have beneficial effects on neuroinflammation and blood-brain barrier dysfunction in the context of TBI [13, 15, 52]. Rodent TBI models result in damage to the BBB by trauma (cell death), inflammation, or ischemia. In this study, we sought to determine the molecular mechanisms of MAGL-mediated BBB protection using animal models which utilize inflammation or ischemia to drive damage to the BBB. Here, we demonstrate that inhibition of MAGL activity can attenuate BBB breakdown, as measured by the extravasation of brain impermeable endogenous proteins or fluorescently labeled tracers, after either inflammatory (LPS) or ischemic (rose bengal) insults. To pressure test the clinical utility of a MAGL inhibitor, we withheld treatment for 6 h and then administered the compound. This time frame is within the required treatment window (within 3–4.5 h of onset of ischemia) for ischemic stroke patients to qualify for tPA treatment for example. Our results demonstrate that inhibition of 2-AG hydrolysis still exerted significant protection of BBB function in animals with delayed treatment.

## Conclusions

Previous work has not looked at the mechanism of the efficacy of endocannabinoid degradation inhibitors [15], or attributed this efficacy in TBI models to enhanced cannabinoid receptor activation (lesion volume and neurodegeneration) through the use of cannabinoid receptor antagonists [52]. Here, we show for the first time that the effect of MAGL inhibition in preserving BBB function is driven through the reduction of arachidonate production and through enhanced cannabinoid signaling depending on the nature of the initial insult. In the inflammation-driven LPS model, co-treatment with CB1 and CB2 antagonists did not significantly reverse the CPD-4645 effect on BBB breakdown, suggesting a less pronounced cannabinoid-dependent mechanism of action in this particular assay. In contrast, in the ischemia-driven photothrombotic model, co-treatment of CPD-4645 with CB1 and CB2 antagonists partially reversed the effect of MAGL inhibition on BBB disruption, suggesting a pronounced, but partial, cannabinoid receptor-dependent mechanism of action. It would therefore be of future interest to analyze expression profiles of the neurovasculature in the presence and absence of cannabinoid receptor antagonists in a more global model of ischemia such as the middle cerebral artery occlusion model, in order to further ascertain whether the BBB pathology in the two models is different or comparable. These results highlight a bi-directional mechanism of action (simultaneous enhancement of cannabinoid signaling through elevation of 2-AG and decrease of AA and downstream eicosanoids) that can achieve therapeutic efficacy through either pathway depending on the nature of the insult.

## Additional files

Additional file 1: **Table S1.** Differentially regulated genes. Differentially regulated genes (increased or decreased by at least twofold) and *p* values for LPS-vehicle vs sham-vehicle and LPS-CPD-4645 vs LPS-vehicle groups. (XLSX 294 kb)

Additional file 2: **Table S2.** Gene ontology analysis. Gene ontology analysis was performed using the DAVID bioinformatics tools. The enriched functional annotation clusters are listed along with *p* values for LPS vs Sham upregulated genes, LPS vs Sham downregulated genes, and CPD-4645 vs vehicle downregulated genes. (XLSX 157 kb)

Additional file 3: **Figure S1.** In vivo selectivity of CPD-4645. Levels of inhibition of MAGL, ABHD6, and FAAH in the brain after 10 mg/kg subcutaneous dose of CPD-4645 at given tie point as determined by activity-based protein profiling using the pan-serine hydrolase probe FP-rhodamine. Full MAGL inhibition was normalized by effect seen at 2 h post-subcutaneous administration of 16 mg/kg JZL-184. (TIFF 1521 kb)

Additional file 4: **Figure S2.** Principal component analysis. Principal component analysis (PCA) for transcriptional signatures demonstrating a distinction between experimental groups. The first two principal components are plotted with the variance explained by each component shown on the axes. (TIFF 1521 kb)
