# Supplementary figures and images for: Inhibition of 2-AG hydrolysis differentially regulates blood brain barrier permeability after injury
Source: J Neuroinflammation. 2018 May 14;15:142. doi: 10.1186/s12974-018-1166-9 (PMC5952841; doi:10.1186/s12974-018-1166-9)

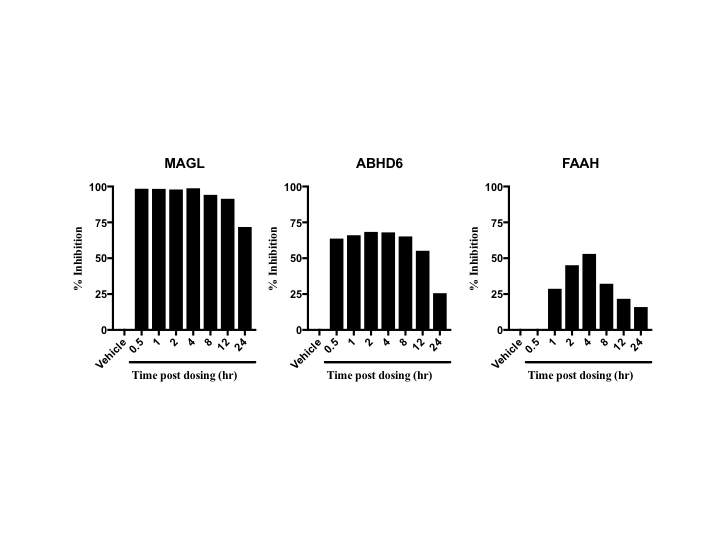

Supplement: Supplementary file 3 — Figure S1. In vivo selectivity of CPD-4645. Levels of inhibition of MAGL, ABHD6, and FAAH in the brain after 10 mg/kg subcutaneous dose of CPD-4645 at given tie point as determined by activity-based protein profiling using the pan-serine hydrolase probe FP-rhodamine. Full MAGL inhibition was normalized by effect seen at 2 h post-subcutaneous administration of 16 mg/kg JZL-184. (TIFF 1521 kb) [file 12974_2018_1166_MOESM3_ESM.tiff]

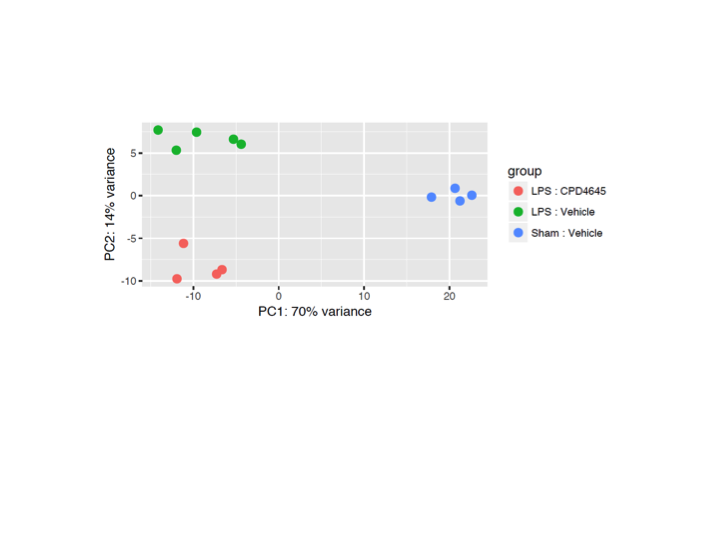

Supplement: Supplementary file 4 — Figure S2. Principal component analysis. Principal component analysis (PCA) for transcriptional signatures demonstrating a distinction between experimental groups. The first two principal components are plotted with the variance explained by each component shown on the axes. (TIFF 1521 kb) [file 12974_2018_1166_MOESM4_ESM.tiff]
